# Nuclear Factor (Erythroid-Derived)-Related Factor 2-Associated Retinal Pigment Epithelial Cell Protection under Blue Light-Induced Oxidative Stress

**DOI:** 10.1155/2016/8694641

**Published:** 2016-09-27

**Authors:** Kei Takayama, Hiroki Kaneko, Keiko Kataoka, Reona Kimoto, Shiang-Jyi Hwang, Fuxiang Ye, Yosuke Nagasaka, Taichi Tsunekawa, Toshiyuki Matsuura, Norie Nonobe, Yasuki Ito, Hiroko Terasaki

**Affiliations:** ^1^Department of Ophthalmology, Nagoya University Graduate School of Medicine, Nagoya 466-8550, Japan; ^2^Laboratory of Bell Research Center-Department of Obstetrics and Gynecology Collaborative Research, Nagoya University Graduate School of Medicine, Nagoya 466-8550, Japan; ^3^Department of Ophthalmology, Shanghai First People's Hospital, Shanghai Jiao Tong University School of Medicine, Shanghai 200240, China

## Abstract

*Purpose*. It is a matter of increasing concern that exposure to light-emitting diodes (LED), particularly blue light (BL), damages retinal cells. This study aimed to investigate the retinal pigment epithelium (RPE) damage caused by BL and to elucidate the role of nuclear factor (erythroid-derived)-related factor 2 (Nrf2) in the pathogenesis of BL-induced RPE damage.* Methods*. ARPE-19, a human RPE cell line, and mouse primary RPE cells from wild-type and* Nrf2* knockout (*Nrf2* ^−/−^) mice were cultured under blue LED exposure (intermediate wavelength, 450 nm). Cell death rate and reactive oxygen species (ROS) generation were measured. TUNEL staining was performed to detect apoptosis. Real-time polymerase chain reaction was performed on* NRF2* mRNA, and western blotting was performed to detect Nrf2 proteins in the nucleus or cytoplasm of RPE cells.* Results*. BL exposure increased cell death rate and ROS generation in ARPE-19 cells in a time-dependent manner; cell death was caused by apoptosis. Moreover, BL exposure induced* NRF2* mRNA upregulation and Nrf2 nuclear translocation in RPE. Cell death rate was significantly higher in RPE cells from* Nrf2* ^−/−^ mice than from wild-type mice.* Conclusions*. The Nrf2 pathway plays an important role in protecting RPE cells against BL-induced oxidative stress.

## 1. Introduction

Age-related macular degeneration (AMD) leads to blindness, accounting for approximately 9% of blindness cases worldwide. There are approximately 30 million patients with AMD, of whom > 0.5 million have become blind [1]. AMD can be divided into two categories: wet and dry AMD [2]. In wet AMD, choroidal neovascularization breaks the retinal pigment epithelium (RPE) through to the neural retina, causing the leakage of fluid, lipids, and blood; these changes lead to fibrous scarring and antivascular endothelial growth factor drugs are approved for wet AMD treatment [3–5]. In dry AMD, progressive geographic atrophy of RPE occurs, followed by severe damage of the photoreceptors. Severe irreversible blindness from AMD is caused by these advanced forms [6, 7].

AMD may have a multifactorial pathogenesis [8] and is characterized by photoreceptor cell death [9–14]. Several factors, such as smoking, obesity, eating habits, and light exposure, particularly blue light (BL) exposure, play important roles in the progression of AMD [15–18]. BL exposure causes an increase in the reactive oxygen species (ROS), which may result in structural damage and decreased viability of retinal cells. It also causes RPE apoptosis via oxidative stress and mitochondrial damage [19–21].

One of the most important antioxidation pathways is the nuclear factor (erythroid-derived)-like 2 (Nrf2) pathway. Nrf2 is a 65 kDa molecule with a basic leucine zipper structure [22]. Without oxidative stress, Nrf2 in its inactive state is bound to Kelch-like ECH-associated protein 1 (Keap1) in the cytoplasm [23]. When cells are exposed to oxidative stress, the active site cysteine residues of Keap1 are oxidized, preventing Keap1 from interacting with* NRF2*. With Nrf2 accumulation in the cytoplasm, Nrf2 moves to the nucleus and binds to the antioxidant response element [24]. Nrf2 also serves as the master regulator of a highly coordinated antioxidant response in RPE cells [25]. Several studies demonstrated that antioxidative factors prevent RPE cells from being damaged by oxidative stress [26–34]. Some antioxidative factors also upregulate Nrf2 signaling. RPE damage may be prevented by these antioxidative factors via the upregulation of Nrf2 signaling. However, little is known regarding whether Nrf2 signaling activation is directly involved in RPE protection. Moreover, to the best of our knowledge, the direct relationship between BL exposure and Nrf2 signaling in RPE cells has not been well elucidated.

*Nrf2* knockout (*Nrf2* ^−/−^) mice have been used in studies on systemic and ocular diseases. In ophthalmology, there are some studies regarding simulating diabetic retinopathy, ischemic retinopathy, and macular degeneration [32, 35–40].* Nrf2* ^−/−^ mice developed ocular pathology similar to AMD [35]. Moreover, primary RPE cells from* Nrf2* ^−/−^ mice are susceptible to oxidative stress [32]. However, to the best of our knowledge, no studies have focused on* Nrf2* ^−/−^ RPE cells exposed to BL. Therefore, in this study, we prepared primary RPE cells from* Nrf2* ^−/−^ mice and investigated the direct involvement of* Nrf2* signaling in BL-induced RPE cell damage.

## 2. Materials and Methods

### 2.1. Cell Culture, Primary Cell Preparation, and BL Exposure

ARPE-19, a human RPE cell line, was purchased from the American Type Culture Collection (Rockville, MD, USA), and primary human RPE (hRPE) cell line was purchased from Lonza (Walkersville, MD, USA). Cells were grown in colorless Dulbecco's modified Eagle's medium (DMEM) premixed with Ham's F-12 (1 : 1 ratio, Sigma-Aldrich) and supplemented with 10% fetal bovine serum and the antibiotics streptomycin/penicillin G (Sigma-Aldrich) [13, 14]. Primary mouse RPE cells were collected from the wild-type and* Nrf2* ^−/−^ mice, as previously described [41, 42]. In brief, mouse eyecups were washed with sterile PBS, and flatmounts were created. The retina was gently removed to allow RPE layer to be on the surface of the flatmount. The RPE eyecups were rinsed in a chelating agent (Versene, Invitrogen), and RPE cells were enzymatically dislodged by 2% Dispase (Roche Diagnostics). Dislodged RPE cells were collected and cultured in DMEM containing antibiotics at 37°C containing 5% CO_2_. Animal studies were approved by the Institutional Animal Care and Use Committee of the Nagoya University Graduate School of Medicine. All procedures involving animals were conducted according to the Association for Research in Vision and Ophthalmology Statement for the Use of Animals in Ophthalmic and Vision Research.* Nrf2* ^−/−^ mice were provided by RIKEN BRC through the National Bio-Resource Project of MEXT, Japan [43]. The cells were cultured in the dark or under BL (Zensui LED Lamp Blue*™*; Zensui Inc., Japan; peak wavelength: 450 *μ*m, 1,200 lux).

### 2.2. LDH Assay and ROS Measurement

The cell death rate was evaluated by measuring the lactate dehydrogenase (LDH) activities using the Cytotoxicity Detection Kit PLUS (Roche Diagnostics, Mannheim, Germany). The supernatant of the culture medium, which contained LDH secreted from dead cells, was collected, followed by the addition of Triton X-100 in the medium to release intracellular LDH from the surviving cells. After measuring the LDH activities in the culture supernatant and medium, the proportions of dead cells among the total cells were calculated. Oxidative stress on BL exposure was evaluated with respect to the amount of ROS, measured using the OxiSelect*™* ROS assay kit (Cell Biolabs. Co., Japan). In brief, after BL exposure, the assay was terminated by adding cell lysis buffer, and fluorescence intensity was measured at 493 nm (ex)/523 nm (em) using a fluorescent plate reader at each time point.

### 2.3. Cell Morphology and TUNEL Staining

Morphological changes of ARPE-19 cells exposed to BL were visualized using a phase-contrast microscope (FSX-100; Olympus, Tokyo, Japan). TUNEL-positive apoptotic cells were detected, as previously described [14, 44]. In brief, after 24 h of BL exposure, the cells were fixed with 2% PFA for 20 min at room temperature on the chambered cell culture slides. The cells were stained with the* In Situ* Cell Death Detection kit (Roche Diagnostics, Mannheim, Germany) and 0.3 mg/mL 4′,6-diamidino-2-phenylindole (Invitrogen, Carlsbad, CA, USA) for 1 h. The stained cells were then observed using a Bio Imaging Navigator fluorescence microscope (BZ-9000; Keyence, Osaka, Japan). The number of TUNEL-positive cells was calculated from images obtained with a 20x lens (537 × 710 *μ*m). The average number of TUNEL-positive cells observed in three independent areas was calculated per well (*n* = number of wells) [14].

### 2.4. Protein and RNA Isolation

For total protein collection, the cultured human and mouse cells were lysed in RIPA buffer (Sigma-Aldrich) with a protease inhibitor cocktail (Roche Diagnostics, Indianapolis, IN, USA). The lysate was centrifuged at 15,000 ×g for 15 min at 4°C, and the supernatant was collected. Protein concentrations were determined using the Bradford Assay Kit (Bio-Rad, Hercules, CA, USA) with bovine serum albumin as the standard. To measure Nrf2 abundance in the nucleus, ARPE-19 cells were treated with NE-PER Nuclear and Cytoplasmic Extraction Kit (Pierce, Rockford, IL, USA) as previously described [41]. For real-time polymerase chain reaction (RT-PCR) analyses, total RNA was purified using the Qiagen RNeasy Mini Kit (Qiagen, Hilden, Germany), according to the manufacturer's protocol; the RNA concentration and quality were assessed using the NanoDrop ND-1000 spectrophotometer (NanoDrop Technologies, Rockland, DE, USA) [13].

### 2.5. Quantitative Reverse Transcription-PCR (RT-PCR)

The total RNA was reverse transcribed using the Transcriptor Universal cDNA Master Kit (Roche Diagnostics), starting with 2 *μ*g of total RNA from each sample [13]. RT-PCR was performed using the Thunderbird Probe qPCR Mix (Toyobo Life Science, Osaka, Japan) and Gene Expression Assay containing primers and an FAM dye-labeled TaqMan probe for detecting human* NRF2* (HS00965961-g1; Applied Biosystems, USA) and eukaryotic 18S rRNA (Hs_99999901_s1; Applied Biosystems) that is available for human 18S rRNA [12]. PCR cycles consisted of a predenaturation step at 95°C for 2 min followed by 40 cycles of denaturing steps at 95°C for 15 s and annealing and extending steps at 60°C for 60 s. The relative expressions of the target genes were determined using the 2^−ΔΔCt^ method.

### 2.6. Western Blotting

Western blotting was performed as previously described [13, 14]. In brief, proteins (50 *μ*g) from ARPE-19 cells were run on SDS precast gels (Wako, Osaka, Japan) and were transferred to PVDF membranes. The transferred membranes were washed with TBS-T (0.05 M Tris, 0.138 M NaCl, 0.0027 M KCl, pH = 8.0, and 0.05% Tween 20; Sigma-Aldrich) and then blocked with 5% nonfat dry milk/TBS-T at room temperature for 2 h. The membranes were then incubated with the rabbit antibody against NRF2 (1 : 100; Santa Cruz Biotechnology) at 4°C overnight. Total protein loading was assessed by immunoblotting using *β*-actin (1 : 3000: Cell Signaling), and nuclear protein loading was assessed by immunoblotting using lamin B (rabbit, 1 : 200; Santa Cruz Biotechnology) [45]. HRP-linked secondary antibody was used (1 : 3,000, Invitrogen) at RT for 1 h. The signal was visualized with enhanced chemiluminescence (ECL plus; GE Healthcare, Piscataway, NJ, USA) and captured using ImageQuant LAS-4000 Imager (GE Healthcare).

### 2.7. Outcomes and Statistical Analysis

Cell death rate, ROS generation, and RT-PCR of Nrf2 mRNA were statistically analyzed using the Mann-Whitney *U* test.* P* values of <0.05 were considered to be statistically significant.

## 3. Results

**Figure 1:**
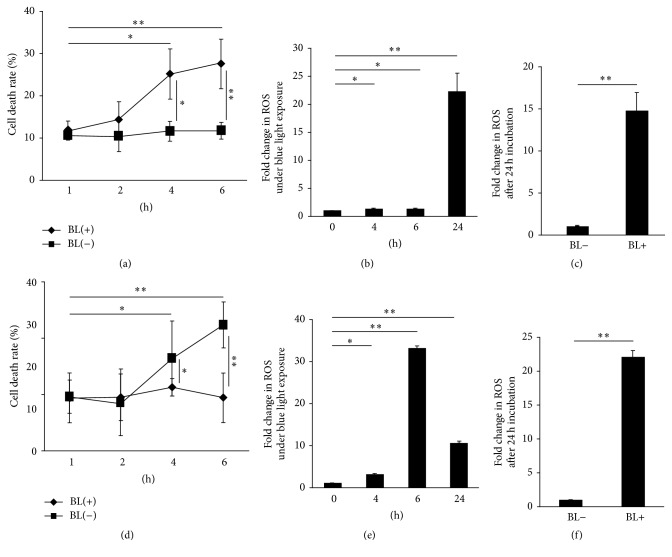
Increases in cell death rate and ROS generation in ARPE-19 cells and human RPE (hRPE) cells with or without BL exposure. (a) BL exposure increased the cell death rate and (b) ROS generation in ARPE-19 cells exposed to BL in a time-dependent manner. (c) ROS reactivity in ARPE-19 cells was significantly higher with BL exposure than without BL exposure 24 h later. (d) BL exposure increased the cell death rate and (e) ROS generation in hRPE cells exposed to BL. (f) ROS reactivity in hRPE cells was significantly higher with BL exposure than without BL exposure 24 h later. ^*∗*^ *P* < 0.05; ^*∗∗*^ *P* < 0.01.

First, we examined whether BL exposure induced ROS generation and RPE cell death. BL exposure caused ARPE-19 cells to release LDH. The values of BL-induced/total LDH were 12.0%  ± 4.2% (*n* = 4), 14.4%  ± 5.9% (*n* = 4), 25.3%  ± 5.8% (*n* = 4), and 27.7%  ± 5.7% (*n* = 4) at 1, 2, 4, and 6 h after BL exposure, respectively. Among these values, there were significant increases at 4 h (*P* = 0.012) and 6 h (*P* = 0.0012) compared with the value at 1 h after BL exposure. In contrast, the values of BL-free/total LDH were 10.6%  ± 3.8% (*n* = 4), 10.6%  ± 2.3% (*n* = 4), 11.6%  ± 2.0% (*n* = 4), and 11.8%  ± 4.8% (*n* = 4) at 1, 2, 4, and 6 h after the onset of treatment, respectively. Among these values, there were also significant differences at 4 h (*P* = 0.014) and 6 h (*P* = 0.0057) compared with those without BL exposure. These findings also showed that ARPE-19 cell death was promoted by BL exposure in a time-dependent manner. This was confirmed by the LDH results from ARPE-19 cells not exposed to BL (Figure 1(a)). BL exposure also induced ROS generation in ARPE-19 cells in a time-dependent manner. ROS generation was significantly higher at 4 h (1.10 ± 0.05, *n* = 5, *P* = 0.027), 6 h (1.32 ± 0.11, *n* = 5, *P* = 0.043), and 24 h (22.4 ± 3.2, *n* = 5, *P* < 0.001) than at 1 h (1.0 ± 0.06, *n* = 5) after BL exposure (Figure 1(b)). Compared with ARPE-19 cells at 24 h after the onset of the treatment without BL exposure (control, 1.00 ± 0.10, *n* = 8), ARPE-19 cells exposed to BL generated significantly higher amounts of ROS (Figure 1(c); 14.8 ± 2.1, *n* = 8, *P* < 0.001). In hRPE cells, the values of BL-induced/total LDH were 9.5%  ± 2.9% (*n* = 4), 8.6%  ± 5.9% (*n* = 4), 16.4%  ± 6.7% (*n* = 4), and 22.3%  ± 5.7% (*n* = 4) at 1, 2, 4, and 6 h after BL exposure, respectively. Among these values, there were significant increases at 4 h (*P* = 0.034) and 6 h (*P* = 0.0011) compared with the value at 1 h after BL exposure. In contrast, the values of BL-free/total LDH were 9.4%  ± 4.4% (*n* = 4), 9.5%  ± 4.1% (*n* = 4), 11.2%  ± 1.6% (*n* = 4), and 9.4%  ± 4.4% (*n* = 4) at 1, 2, 4, and 6 h after the onset of treatment, respectively. Among these values, there were significant differences at 4 h (*P* = 0.048) and 6 h (*P* < 0.001) compared with those without BL exposure. These findings also showed that hRPE cell death was promoted by BL exposure in a time-dependent manner. This was confirmed by LDH results from hRPE cells not exposed to BL (Figure 1(d)). BL exposure also induced ROS generation in hRPE cells. ROS generation was significantly higher at 4 h (3.23 ± 0.07, *n* = 8, *P* < 0.001), 6 h (33.28 ± 0.49, *n* = 8, *P* < 0.001), and 24 h (10.54 ± 0.41, *n* = 8, *P* < 0.001) than at 1 h (1.0 ± 0.06, *n* = 8) after BL exposure (Figure 1(e)). Compared with hRPE cells at 24 h after the onset of the treatment without BL exposure (control, 1.00 ± 0.07, *n* = 8), hRPE cells exposed to BL generated significantly higher amounts of ROS (Figure 1(f); 22.12 ± 0.91, *n* = 8, *P* < 0.001).

**Figure 2:**
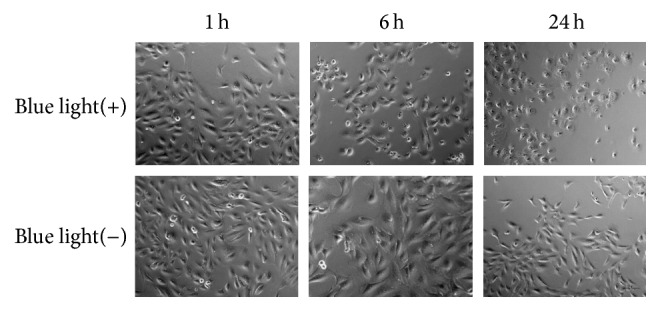
Morphological changes of ARPE-19 cells with or without blue light (BL) exposure. ARPE-19 cells changed to an oval shape and shrank upon BL exposure in a time-dependent manner (1, 6, and 24 h), whereas only minor changes were shown in those without BL exposure. Scale bar = 100 *μ*m.

**Figure 3:**
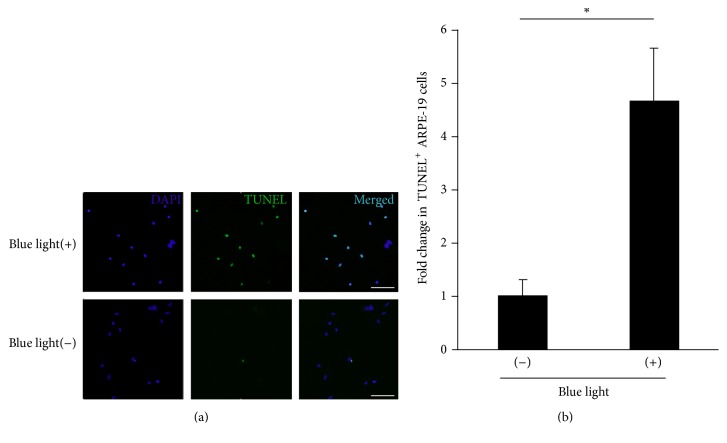
Detection of apoptosis in ARPE-19 cells at 24 h with or without BL exposure. (a) TUNEL (green) staining showed that, 24 h after BL exposure, ARPE-19 cells showed TUNEL positivity, reflecting apoptosis, by BL exposure. (b) There was a significant increase (4.67-fold) in the number of TUNEL-positive RPE cells with BL exposure compared to those without BL exposure. DAPI indicates the nucleus. Scale bar = 100 *μ*m. ^*∗*^ *P* < 0.01.

Moreover, we examined the morphological change of ARPE-19 cells on BL exposure. Under normal condition, ARPE-19 cells showed a spindle shape. However, on BL exposure, these cells shrank and changed to an oval shape. These findings indicated that BL exposure decreased the viability of ARPE-19 cells as previously described (Figure 2) [46]. To clarify more precisely the mechanism of ARPE-19 cell death by BL exposure, we performed TUNEL staining of ARPE-19 cells with and without BL exposure. This staining revealed that numerous ARPE-19 cells were TUNEL-positive at 24 h after BL exposure. These findings indicated that BL exposure-induced ARPE-19 cell death mostly involved apoptosis (Figure 3).

**Figure 4:**
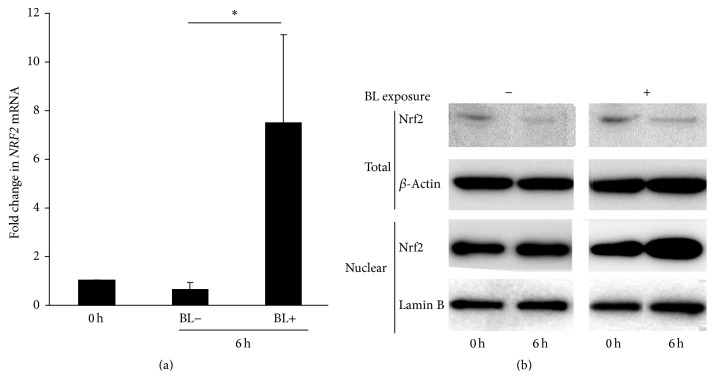
Nrf2 abundance in ARPE-19 cells with blue light (BL) exposure. (a) BL exposure increased* NRF2* mRNA level in ARPE-19 cells and showed a significant difference at 6 h compared with that without BL exposure. (b) Nrf2 protein in the total ARPE-19 cells was decreased at 6 h of exposure, although Nrf2 protein in the nucleus was increased at 6 h of exposure. ^*∗*^ *P* < 0.05.

**Figure 5:**
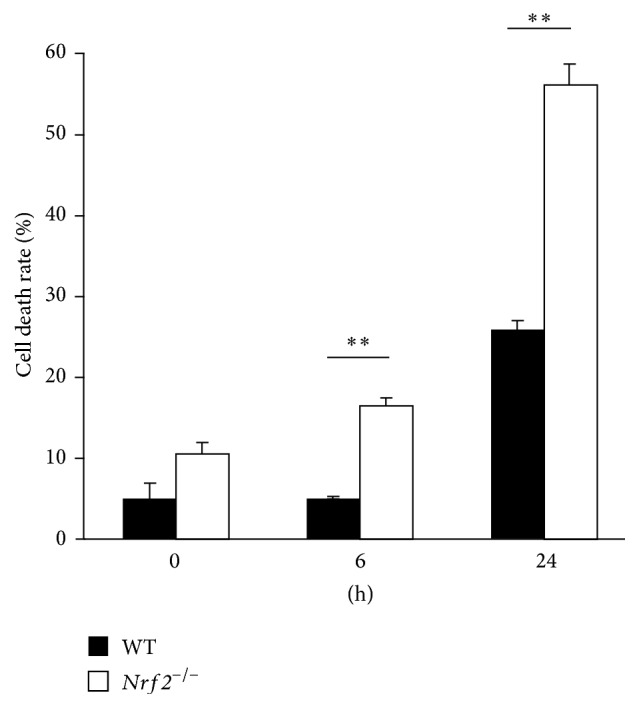
Increased cell death in* Nrf2* ^−/−^ RPE cells under blue light (BL) exposure. BL exposure induced more cell death in* Nrf2* ^−/−^ RPE cells than in wild-type RPE cells. ^*∗∗*^ *P* < 0.01.

To investigate the involvement of* NRF2* in the effect of BL exposure on ARPE-19 cells, we examined mRNA level and* NRF2* protein expression of ARPE-19 cells with and without BL exposure. Compared with the mRNA level of ARPE-19 cells at the onset of treatment (control, 1.00 ± 0.09, *n* = 2), the level at 6 h after BL exposure was significantly higher (Figure 4(a); 7.24 ± 3.74, *n* = 8, *P* = 0.042).* NRF2* mRNA level was increased by BL exposure, whereas it did not significantly increase in the absence of BL exposure from 0 to 6 h after the onset of treatment. Nrf2 protein expression from total ARPE-19 cells appeared to be decreased by BL exposure (Figure 4(b)). In an active state, Nrf2 showed nuclear translocation. Therefore, we separately obtained Nrf2 protein from the nucleus of ARPE-19 cells. Nrf2 protein was abundantly expressed in the ARPE-19 nucleus (Figure 4(b)). These results showed that, as a result of BL exposure, Nrf2 in ARPE-19 cells was activated. To shed more light on the importance of Nrf2 activation in BL exposure-induced ARPE-19 cell damage, we collected primary RPE cells from* Nrf2* ^−/−^ and wild-type (*Nrf2* ^*+/+*^) mice and compared their cell death rates on BL exposure. Under these conditions, death rates of RPE cells from* Nrf2* ^−/−^ mice were 16.4 ± 1.1 (*n* = 6) and 56.1 ± 2.8 (*n* = 6) at 6 and 24 h after exposure, respectively. In contrast, death rates of RPE cells from wild-type mice were 5.0 ± 0.1 (*n* = 6) and 25.7 ± 1.4 (*n* = 6) at 6 and 24 h after exposure, respectively. There were significant increases in the death rate in* Nrf2* ^−/−^ mouse RPE cells at 6 h (*P* < 0.001) and 24 h (*P* < 0.001) compared with those in wild-type mice (Figure 5). These findings indicated that Nrf2 plays an important role in blocking cell death caused by BL exposure-induced ROS generation.

## 4. Discussion

Visible light exposure-induced damage in retinal cells occurs through type I (free radical) and type II (oxygen-dependent) mechanisms. Free radicals induce cells to undergo necrosis, whereas oxygen-dependent mechanisms induce them to undergo apoptosis [47]. The mechanism of AMD was considered to involve oxidative stress caused by several factors driving RPE cells to die via apoptosis. BL exposure induces ROS generation, damaging mitochondrial DNA and cell structure; then, RPE cells are forced to enter an apoptotic state [15–17]. In this study, we demonstrated that BL exposure increased ROS generation in RPE cells and induced cell death in a time-dependent manner. In addition, TUNEL staining suggested that apoptotic RPE cell death was caused by BL exposure. To an extent, these findings simulate the pathogenesis of AMD.

ARPE-19 cells have some differences from primary hRPE cells in terms of promoter strength [48], proliferation, and cell death [49]. Previous studies have demonstrated that ARPE-19 cells are stronger and tolerable for oxidative stress compared with primary hRPE cells. In the present study, cell death rate and ROS of primary hRPE cells at 6 h of BL exposure (Figures 1(d) and 1(e)) were higher than those of ARPE-19 cells (Figures 1(a) and 1(b)). It has been previously demonstrated that primary hRPE cells are more sensitive than ARPE-19 cells against BL exposure-derived oxidative stress. On the other hand, ROS generation at 24 h was lower than that at 6 h in primary hRPE cells. This is possibly because primary hRPE cells were killed by 24 h BL exposure, and lower ROS generation was measured from the remaining (smaller number of) cells. Although both cells were damaged by BL exposure-derived ROS in a time-dependent manner, we considered that the use of these cells for this BL exposure study design is suitable.

The Nrf2 pathway is one of the most important pathways for protecting cells against oxidative stress [22]. Without oxidative stress, Nrf2, in its inactive state, is kept in the cytoplasm [23]. When cells are exposed to oxidative stress, this Nrf2 is released from Keap1 and moves to the nucleus where it functions in antioxidative protective mechanisms [24]. Other cells, such as epidermoid carcinoma cells [50] and retinal ganglion cells [51], also showed increased Nrf2 protein expression on BL exposure* in vitro*. These reports suggested that BL exposure increased ROS generation, activated* Nrf2* signaling, and reduced cell viability in a time-dependent manner. In our study, BL exposure of RPE cells increased ROS generation and apoptotic cell death rate in a time-dependent manner. In addition,* Nrf2* mRNA level and Nrf2 protein expression in the nucleus were increased in RPE cells in a time-dependent manner. These findings indicated that BL exposure induces the upregulation of ROS and Nrf2, which are involved in antioxidative protective mechanisms as previously reported.

If the* Nrf2* pathway does not properly function, antioxidative protection would be weaker and oxidative stress would cause severe cell damage.* Nrf2* ^−/−^ mice developed ocular pathology similar to the cardinal features of human AMD; deregulated autophagy is a likely mechanistic link between oxidative injury and inflammation [35].* Nrf2* ^−/−^ RPE cells are susceptible to oxidative stress induced by t-butylhydroperoxide [32]. However, these studies did not use light exposure as an oxidative stressor. In dermatology, some studies revealing the relationship between* Nrf2* ^−/−^ cells and light exposure have been published [52–55]. Ultraviolet light-irradiated* Nrf2* ^−/−^ cells exhibited accelerated photoaging, resulting in the necrosis of irradiated cells, inflammatory cell infiltration, TUNEL-positive apoptotic cell formation, and the accumulation of oxidative DNA products, which are caused by oxidative stress. In this study,* Nrf2* ^−/−^ RPE cells died at rates of 10.5%, 16.4%, and 56.1% at 0, 6, and 24 h of BL exposure, respectively. This rate of cell death at 0 h tended to be higher than that of wild-type RPE cells, and the rates at 6 and 24 h were significantly higher than those of the wild-type RPE cells. These findings suggest that* Nrf2* ^−/−^ RPE cells are weaker than wild-type RPE cells, and* Nrf2* signaling plays a key protective role against BL-induced oxidative stress.

Previous studies demonstrated that some materials, such as polyphenol, salvianolic acid, 4-acetoxyphenol, 17-beta-estradiol triterpenoid RTA-408, pinosylvin, alpha-mangostin, and coconut water, protect cells against oxidative stress via* Nrf2* signaling. They showed that these materials upregulate Nrf2 protein expression and concluded that these materials protect cells against oxidative stress by increasing Nrf2 protein expression. However, it is unknown whether* Nrf2* protein expression is increased by these materials to rescue cells from oxidative stress or whether the expression is secondarily increased in a manner independent of these materials [26–34]. These materials would be expected to stimulate the* Nrf2* pathway and increase* Nrf2* protein expression to protect cells against oxidative stress.

A limitation of this study is that we examined only an* in vitro* biological change. A previous study used* Nrf2* ^−/−^ mice subjected to direct light exposure* in vivo* and revealed tissue change caused by oxidative stress [54]. Studying the effect of BL exposure on living* Nrf2* ^−/−^ mice will provide us with more precise information regarding the adverse effects of BL exposure on the eyes and the importance of the Nrf2 pathway in protecting eyes during BL exposure.

In conclusion, BL exposure induced ROS generation and caused cell death via apoptosis in RPE cells. The* Nrf2* pathway plays a protective role against oxidative stress on BL exposure. Our findings in this study are meaningful for demonstrating the direct relationship between BL exposure and* Nrf2* signaling, as proved by* Nrf2* ^−/−^ in RPE cells.
